# High-Throughput Analysis of Amino Acids for Protein Quantification in Plant and Animal-Derived Samples Using High Resolution Mass Spectrometry

**DOI:** 10.3390/molecules26247578

**Published:** 2021-12-14

**Authors:** Priyanka Reddy, Aaron Elkins, Joe Panozzo, Simone J. Rochfort

**Affiliations:** 1 Agriculture Victoria, AgriBio, Centre for AgriBioscience, Bundoora, VIC 3083, Australia; priyanka.reddy@agriculture.vic.gov.au (P.R.); aaron.elkins@agriculture.vic.gov.au (A.E.); 2 Centre for Agricultural Innovation, University of Melbourne, Parkville, VIC 3010, Australia; joe.panozzo@agriculture.vic.gov.au; 3 Agriculture Research Victoria, 110 Natimuk Road, Horsham, VIC 3400, Australia; 4 School of Applied Systems Biology, La Trobe University, Bundoora, VIC 3083, Australia

**Keywords:** hydrolysis, pulse, lentil, bovine serum albumin (BSA), LC–MS

## Abstract

Current methods for measuring the abundance of proteogenic amino acids in plants require derivatisation, extended run times, very sensitive pH adjustments of the protein hydrolysates, and the use of buffers in the chromatographic phases. Here, we describe a fast liquid chromatography–mass spectrometry (LC–MS) method for the determination of amino acids that requires only three steps: hydrolysis, neutralisation, and sample dilution with a borate buffer solution for pH and retention time stability. The method shows excellent repeatability (repeated consecutive injections) and reproducibility (repeated hydrolysis) in the amino acid content, peak area, and retention time for all the standard amino acids. The chromatographic run time is 20 min with a reproducibility and repeatability of <1% for the retention time and <11% for the peak area of the BSA and quality control (QC) lentil samples. The reproducibility of the total protein levels in the hydrolysis batches 1–4 was <12% for the BSA and the lentil samples. The level of detection on column was below 0.1 µM for most amino acids (mean 0.017 µM).

## 1. Introduction

The amino acid composition of pulses is an important factor contributing to the nutritional quality of proteins. The assessment of amino acids can guide the selection of the germplasm within plant breeding programs; however, due to the current time-consuming sample preparation techniques, it is often not assessed. Therefore, protein percentage is often used as a surrogate for nutritional quality.

The high levels of protein in pulses make it a healthy alternative to meat-derived protein. Moreover, the environmental pressures on sustainable farming have spurred research activities in plant-based proteins and in improving the quality of the protein in pulses. However, to increase the appeal of pulses as an alternative protein source to incorporate into the human diet, the molecular phenotypes must be assessed so to underpin the genomic approaches to improve the breeding of pulses for improved human health.

Commonly used methods for protein quantification of total protein in foods include UV spectroscopic measurements or more sensitive dye-binding assays that use colorimetric and fluorescent-based detection that rely on inexpensive equipment [1,2,3]. Other developed methods that meet the required sensitivity and automation include the isotope dilution mass spectrometry (IDMS) of peptides released by the enzymatic digestion of proteins [4]. Many LC–MS methods for quantitation have also reported and require the liberation of the amino acids of proteins for individual quantification without the associated degradation and derivatisation [5]. For example, a commonly used LC-QQQ MS method reported the use of a fluorescent Aqc tag that confers hydrophobicity to the polar target molecule, allowing for the separation of hydrophilic amines, and which is also the identifier, and the amino quinoline moiety (Amq) that is bound to a corresponding fragment ion. This is due to the improved sensitivity of using a liquid chromatography with a triple-quadrupole mass spectrometry (LC-QQQ MS) aminoquinolyl-N-hydroxysuccinimidyl carbamate (AQC).

The previously undertaken analysis of proteogenic amino acids in plant materials involved very sensitive pH adjustments, derivatisation [5,6], extended run times (45–60 min) [7], and the use of complex buffers in mobile phase solutions (with a pH-adjusted aqueous phase) with ion exchange chromatographic techniques [8]. Spectral deconvolution with LC/MS systems is expanding in their mass accuracy capabilities; however, the challenge with proteogenic and nonproteogenic amino acid LC/MS quantification is the isobars or the interrelated compounds with an overlapping isotopologue. For example, (1) leucine, isoleucine, (2) asparagine, aspartate, (3) glutamate, and glutamine. Although there are many LC–MS methods that have been reported, the setup is often nonconventional and requires complex sample preparation and chromatographic systems, as described above. In addition, the retention time stability is critical in liquid chromatography systems and is easily altered with pH instability. Thus, the neutralisation of pH-sensitive hydrolysates can be challenging, and most methods that require derivatisation or buffers in the mobile phases are conducive to pH stability.

Here we provide a three-step sample preparation method to assess the protein levels in lentils involving (1) acid hydrolysis (2), neutralisation, and (3) the dilution of the hydrolysate with a pH buffer. The method offers low-level detectability and an extended dynamic range and is analysed on a high-throughput reversed-phase chromatography system that does not require clean up and that is coupled to a high-resolution mass spectrometer. We report for the first time extensive data on the repeatability of the hydrolysis methods and their injection, retention times, and peak area data that provides a robust and accurate measurement of amino acids using plant (lentil) and animal-derived bovine serum albumin (BSA) samples.

## 2. Results and Discussion

**Figure 1:**
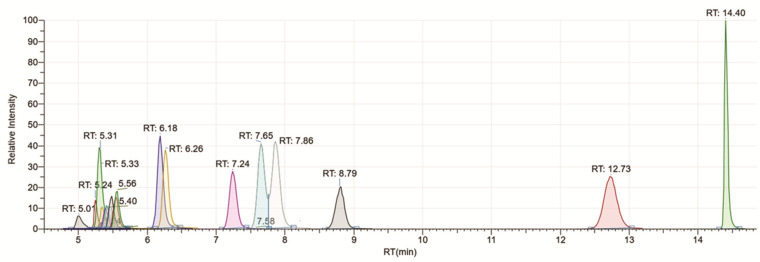
Extracted ion chromatogram (EIC) showing separation of 20 proteogenic amino acids using a C18 reversed phase column with a linear gradient of 2–100% solvent B over 20 min.

To accurately quantify the 20 proteogenic amino acids, chromatographic resolution was achieved on a Polar RP C18 column using a linear gradient of water and acetonitrile. The 20 min LC method was developed with standard RP solvents, including water (0.1% formic acid) and acetonitrile (0.1% formic acid), and a linear gradient of 2% acetonitrile to 100% acetonitrile, which resulted in the chromatographic separation of the isobars, leucine (Leu), and isoleucine (ILeu), as well as amino acids such as asparagine and aspartic acid [9] that are one mass unit apart. Unlike the other high-throughput methods that require buffers in their mobile phases or pH adjustments in their solvents such as HILIC systems, our method can be adapted to the existing reverse phase liquid chromatography systems without requiring clean-up time. The extracted ion chromatogram (EIC) of the 20 amino acids is shown in Figure 1. The extraction of the accurate mass with a 5 ppm tolerance resulted in the spectral deconvolution of the coeluting amino acids and reduced interferences from the impurities.

**Figure 2:**
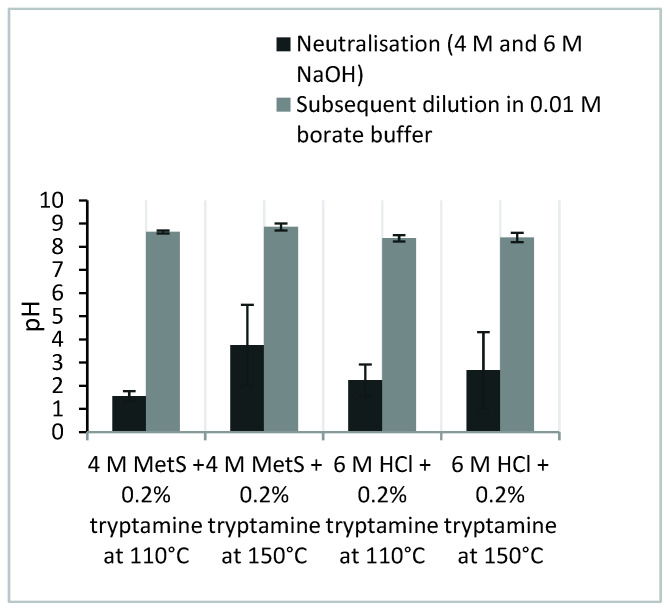
The pH stability achieved by 1:10 dilution with 0.01 M borate buffer following neutralisation with NaOH (4 M or 6 M) (*n* = 5).

The neutralisation of the hydrolysate and the pH sensitivity is commonly reported; however, in previous studies the use of complex derivatisation and buffered mobile phases could stabilise the pH. Our method does not require these additional steps. A simple dilution of the partly neutralised hydrolysate with 0.01 M borate buffer solution results in a stable pH (Figure 2), and thus the reproducible retention times (0.1–0.2%) (Table 1) of the amino acids in the LC–MS analysis. The extracted ion chromatograms (EIC) of the individual amino acids and the total ion chromatogram (TIC) of the standard are provided in Supplementary Figure S1.

Deuterated internal standards are generally used to account for the matrix suppression effects in the sample, and thus to achieve absolute quantification. External standards tend to underestimate the levels present in the samples, as they are not present in a complex matrix or an equivalent matrix to the samples. However, in the crop improvement programs, the levels of the proteogenic amino acids are quantitated across the samples with identical matrices. Thus, in our studies the internal standards were not utilised for the absolute quantitation as the relative levels across the series were adequate for performing the genomic selection. If, however, the levels in the different biological samples need to be compared or the absolute quantitation is required (e.g., for plant and animal tissues), then a commercially available deuterated amino acid mix could be spiked into each of the matrix types and multiple calibration curves could be generated to account for the matrix suppression.

### 2.1. Protein Hydrolysis

Preliminary hydrolysis methods were investigated with the BSA and lentil samples using two oxidising agents, including 6 M HCl or 4 M methane sulfonic (MetS), and 0.2% tryptamine was added as a protective antioxidant [10]. These methods were evaluated at 110 °C for a duration of 22 h or 150 °C for 2 h. The lentil and the BSA protein standards were used to evaluate the four different methods (Table 1). The 4 M MetS hydrolysis method shows a superior recovery of all the amino acids compared to the 6 M HCl method, irrespective of the sample type, and 6 M HCl showed relatively inconsistent recoveries of 75–63% in lentil QC 1 and 95–91% in lentil QC 2. However, in the BSA, 6 M HCl showed poorer (85–81%) recovery when compared to 4 M MetS. The individual amino acid content for lentil QC 1 and lentil QC 2 for each of the methods is provided in Supplementary Tables S1 and S2.

In the BSA, Trp was completely degraded in both of the methods that used 6 M HCl for the hydrolysis, which is reported to occur in carbohydrate-rich samples [9] (Supplementary Table S3). The labile amino acids cysteine (Cys), methionine (Met), and tryptophan (Trp) are prone to degradation, and the losses of Cys were significant in 6 M HCl. For the purposes of the genome-wide association studies (GWAS), the relative concentration of the proteogenic amino acids in a population using an untargeted metabolomics strategy is an adequate approach for the genomic selection. Thus, the extent of the degradation of the free amino acids using the pre- and post-hydrolysis quantitative values was not explored in this study. Deamidation post-hydrolysis can occur to susceptible amino acids, including asparagine and glutamine, that are released from the peptides, but does not have a major impact on the total protein levels as they rearrange to form the closely related amino acids aspartate and glutamate [8].

### 2.2. Method Validation

Here, the LOQ is the smallest concentration of a compound that can be reliably and repeatably quantified. Linear regression gave the best fit and is the optimal point between the LOQ and the highest concentration that results in very good correlation coefficient (R^2^) values equal to or above 0.99 (Table 2). This method is applicable to a wide linear dynamic range for the 20 amino acids, with a limit of quantitation for most compounds <0.1 μM. The LOD is measured as the smallest concentration that can be observed and reliably detected with a signal-to-noise (S/N) ratio above three in our system. The detection limit of 0.001–0.1 ppm for the developed method is consistent with that obtained from high-resolution mass spectrometers (Table 2) [11,12].

This method has been applied to the quantitation of proteogenic amino acids for molecular phenotyping and shows excellent repeatability (repeated consecutive injections) and reproducibility (repeated hydrolysis), calculated as relative standard deviation (RSD) values for the amino acid content, the peak area, and the retention time for all the standard amino acids. The overall average RSD for the injection repeatability for the total protein content in the BSA (2.3–8.2%) and the lentil samples (1–3.7%) is <10% (Table 3). The overall average RSD for the reproducibility for the amino acid content in the BSA is 9.7% and the lentil samples is 12.2% (Table 3). Similarly, the overall reproducibility and the injection repeatability in the peak areas for the BSA (10.7%; 3–10%) and the lentils (10.7%; 1–3%) indicated a good instrument stability (Table 4). The retention time shows <1% injection repeatability and an overall reproducibility, indicating that a stable pH (Table 4) is not only maintained but reproduced with repeated hydrolysis.

The reproducibility and repeatability of the peak areas for the individual amino acids showed little variation from batch 1 to 4 (Table 4), with the exception of the labile amino acids cystine and methionine. Despite tryptophan also being susceptible to degradation, it performed well in the BSA batches (11.80%).

The injection repeatability for the lentil and the BSA for each amino acid for both the peak area and the amino acid content was comparable to the previous reports [1,3,4]. Although the variation in the overall reproducibility is observed for the individual amino acids, which is in part due to the low concentrations, the variation in the total protein content was not significantly impacted. To our knowledge, the reproducibility in hydrolysis, particularly for the duration of 4 weeks, has not been reported. It also provides confidence on the shelf life and the stability of the reagents for the duration of the testing period.

Prior to the data analysis, the individual samples were checked for column pressures, variations of the PBQC, and the retention times for the instrument stability along the batch (Table 5). The internal standard d-valine was used to account for the sample preparation variation, and the instrument repeatability was found to be 0.3–4% for the lentils and 1–15.5% for the BSA (Table 3) within the individual batches. The reproducibility of the internal standard d-valine for the BSA is 14.5% and the lentils is 5.0%.

The deuterated internal standard ^13^C–^15^N-valine showed good repeatability and reproducibility for the lentil (0.3–3.4%; 5%) and the BSA (1.0–15.5%; 14.5%) samples. The ion suppression effect of the matrix on the detectability of the internal standard was expressed as a percentage of the spiked amount (1 ug/mL) relative to the detected levels. In the BSA samples, a 70–87% (Supplementary Table S5) recovery was achieved, and similarly, the lentils (Supplementary Table S4) showed a 74–87% recovery. For the absolute quantitation, the deuterated standards for the individual amino acids could be prepared in the corresponding matrix.

The data show that a reliable quantitative approach for protein analysis can be achieved in lentils and animal biological samples with a simple hydrolysis, neutralisation, and borate dilution method coupled to a reversed phase LC–MS profiling. Although the use of buffers in mass spectrometry usually contributes to ion suppression, the overall detectability for all the amino acids remained comparably low and the source contamination for the duration of the experiment was negligible. However, to further improve the sensitivity of the amino acids, and thus reduce the observed error, the investigation of other buffer solutions could be explored. It is also recommended that, despite the excellent injection repeatability observed in this study, further method development would be required to protect the labile amino acids from degradation and oxidation so to improve the overall hydrolysis reproducibility.

## 3. Materials and Methods

### 3.1. Samples

Lentils (Lens culinaris) with orange-red cotyledon and grey seedcoat were sourced from Agriculture Victoria plant breeding trials grown at Mallala, South Australia in 2019. Bovine serum albumin (BSA) protein standard was obtained from Sigma-Aldrich (St. Louis, MO, USA).

### 3.2. Standards

An amino acid stock solution of 1 mg/mL was prepared using 0.1 N HCl. Twenty amino acids were accurately weighed (50 ± 0.1 mg) and dissolved in 0.1 N HCl in a 50 mL volumetric flask, resulting in a 1 mg/mL stock solution. Sonication was required for the solute to dissolve into solution. The amino acid stock solution included alanine (Ala), asparagine (Asn), aspartic acid (Asp), arginine (Arg), cysteine (Cys), glutamic acid (Glu), glutamine (Gln), glycine (Gly), histidine (His), isoleucine (Ile), leucine (Leu), lysine (Lys), methionine (Met), phenylalanine (Phe), proline (Pro), serine (Ser), threonine (Thr), tryptophan (Trp), tyrosine (Tyr), and valine (Val). Series dilution was performed, and standard solutions of 0.0001, 0.01, 0.05, 0.1, 0.5, 1, 2.5, 5, and 10 µg/mL were prepared in 0.01 M borate buffer. All amino acids standards were purchased from Sigma-Aldrich (St. Louis, MO, USA).

### 3.3. Sample Preparation

The lentil cotyledons were transferred into 15 mL polycarbonate tubes with 2/8″ and 1/8″ stainless steel grinding balls. Sample tubes were placed into 12-well blocks on the Geno/Grinder 2010 (SPEX Sample Prep, Metuchen, NJ, USA), and the cotyledons were homogenised at 1550 rpm for 2 min. Two separate QC samples were prepared of varying lentil compositions, named QC 1 and QC 2. The homogenised powder of each was kept sealed at room temperature until ready to be weighed.

### 3.4. Amino Acid Quantitation Pre-Hydrolysis

Lentil samples were accurately weighed (50 ± 0.2 mg) into a 2 mL microcentrifuge tubes. Lentil powder was extracted using a 4:1 (*v*/*v*) MeOH/H_2_O monophasic methanolic extraction. Briefly, 1 mL of methanol solution was added to the lentil powder and vortexed on a plate vortex. Samples were sonicated for 5 min and centrifuged for 5 min at 10,000 rpm (9503× *g*). A 100 μL aliquot was transferred into HPLC vials containing inserts ready for LC–MS analysis of polar metabolites.

### 3.5. Lentil Hydrolysis

To investigate the four hydrolysis methods for lentils, a total of 100 samples (20 samples with 5 replicates) were accurately weighed (50 ± 0.1 mg) into a 5 mL hydrolysis vial (Wheaton Industries, Millville, NJ, USA) with a screw cap, and 2 mL of 6 M hydrochloric acid or 4 M methane sulfonic acid 0.2% *w*/*v* tryptamine was added. The headspace was purged with nitrogen prior to heating to prevent oxidation. The vial was screwed tightly and placed in a heating block at 110 °C for 22 h or 150 °C for 60 min. After hydrolysis, the samples were allowed to cool to room temperature and then neutralised with 1.9 mL 6 M or 4 M sodium hydroxide and vortexed for 30 s. The exothermic reaction heated up the vials and thus were left to cool. Approximately 1 mL of the hydrolysate was removed and centrifuged at 13,000 rpm for 15 min before 100 µL was aliquoted into a clear HPLC vial containing 890 µL of 0.01 M borate buffer (pH = 9.32). An additional 10 µL of 100 µg/mL solution of ^13^C–^15^N-labelled valine obtained from Sigma-Aldrich (St. Louis, MO, USA) was spiked into samples post-hydrolysis as a measure of LC–MS reproducibility.

### 3.6. Liquid Chromatography–Mass Spectrometry (LC–MS) Analysis

All samples were analysed on a Vanquish Ultra-High Performance Liquid Chromatography (UHPLC) system (Thermo Fisher Scientific, Bremen, Germany) with a binary pump, autosampler, and temperature-controlled column compartment coupled with a QExactive (QE) Plus mass spectrometer (Thermo Fisher, Waltham, MA, USA; Thermo, Bremen, Germany) detector. The Thermo Fisher QExactive Plus mass spectrometer was set at FT switching positive and negative mode over a mass range of 70–1200 amu with the resolution set at 35,000 and the AGC target set to 3 × 10^6^ ions. Nitrogen was used as the sheath, the auxiliary and sweep gas at a flow rate of 28, 15, and 4 L/min, respectively, and the spray voltage was set at 3600 V (positive) and 3300 V (negative). Samples were randomized, and blanks were run every 5 samples. A QC was run every 10 samples. Prior to data acquisition, the system was calibrated with a Pierce^®^ LTQ Velos ESI Positive and Negative Ion Calibration Solution (Thermo Fisher Scientific™). Mass spectrometry data was acquired using a Thermo Xcalibur V. 2.1 (Thermo Fisher Scientific Inc., Waltham, MA, USA). Quantitative analysis was conducted using the LCQUAN™ Quantitative Software (Thermo Fisher Scientific™).

Amino acids were eluted from the column (Phenomenex 250 × 4.6 mm 4 µm Synergi Polar-RP HPLC column) using a gradient mobile phase, A (0.1% formic acid: H_2_O), and B (0.1% formic acid: acetonitrile) at 0.5 mL/min with 98% A to 0% A over 20 min with a linear gradient.

## 4. Conclusions

The three-step method of hydrolysis, neutralisation, and sample dilution with a borate buffer solution for pH and retention time stability allows for quantitative protein analysis on lentil and animal biological samples with excellent reproducibility and repeatability. Furthermore, the sample and reagent stability over the duration of the 4-week acquisition period allows for the quantification of large numbers of samples. The untargeted measurement on a high-resolution QExactive Plus mass spectrometer allows for accurate mass identification and deconvolution with measurements of reliable repeatability and reproducibility. This method can be readily adapted to other biological specimens using many analytical platforms, such as QqTOF and QqQ coupled to a normal LC or UPLC.

## Figures and Tables

**Table 1:** Relative levels of total protein in four hydrolysis methods.

Column	4 M MetS + 0.2% Tryptamine at 110 °C			4 M MetS + 0.2% Tryptamine at 150 °C			6 M HCl + 0.2% Tryptamine at 110 °C			6 M HCl + 0.2% Tryptamine at 150 °C		
Lentil QC 1 (*n* = 5)	100.00	±	1.32	91.52	±	2.59	74.79	±	5.31	62.53	±	4.14
Lentil QC 2 (*n* = 5)	101.55	±	0.62	96.74	±	2.01	94.63	±	0.89	91.12	±	0.58
BSA (*n* = 2)	100.00	±	0.27	98.74	±	3.04	84.66	±	9.04	81.09	±	26.30

% Relative quantitation of 4 M MetS + 0.2% tryptamine at 110 °C.

**Table 2:** LOD, linearity range (≤5 ppm), and retention time of quantitated amino acids.

Compounds	Ions Quantified	Ion Extraction Window (*m*/*z*)	RT (min)	Linearity (ppm)	LOD (µg /mL)	R^2^
Lysine	[M + H]^+^	147.11–147.12	5.01	0.01–10.0	0.001	0.9998
Histidine	[M + H]^+^	156.07–156.08	5.24	0.05–10.0	0.001	0.9986
Cystine *	[M + H]^+^	241.02–241.04	5.26	0.1–10.0	0.01	0.9995
Arginine	[M + H]^+^	175.11–175.12	5.29	0.1–10.0	0.001	0.9995
Glycine	[M + H]^+^	76.03–76.05	5.32	0.25–10.0	0.1	0.9997
Serine	[M + H]^+^	106.05–106.06	5.33	0.1–10.0	0.001	0.9995
Aspartic acid	[M + H]^+^	134.04–134.05	5.38	0.1–10.0	0.001	0.9956
Asparagine	[M + H]^+^	133.06–133.07	5.40	0.1–10.0	0.01	0.9998
Alanine	[M + H]^+^	90.05–90.06	5.45	0.1–10.0	0.001	0.9997
Threonine	[M + H]^+^	120.06–120.07	5.47	0.05–5.0	0.001	0.9991
Glutamic acid	[M + H]^+^	148.06–148.07	5.53	0.05–5.0	0.1	0.9991
Glutamine	[M + H]^+^	147.07–147.08	5.52	0.1–10.0	0.1	0.9996
Proline	[M + H]^+^	116.07–116.08	6.16	0.01–10.0	0.001	0.9980
Valine	[M + H]^+^	118.08–118.09	6.25	0.01–10.0	0.001	0.9972
Methionine	[M + H]^+^	150.05–150.06	7.22	0.001–10.0	<0.001	0.9995
Isoleucine	[M + H]^+^	132.10–132.11	7.66	0.01–10.0	0.001	0.9986
Leucine	[M + H]^+^	132.10–132.11	7.87	0.1–10.0	0.001	0.9982
Tyrosine	[M + H]^+^	182.08–182.09	8.79	0.01–10.0	0.001	1.0000
Phenylalanine	[M + H]^+^	166.09–166.09	12.71	0.01–10.0	0.001	1.0000
Tryptophan	[M + H]^+^	205.09–205.10	14.35	0.01–10.0	0.01	0.9998

* Cysteine was detected as cystine.

**Table 3:** The average relative standard deviations (RSD) of amino acid content of BSA and lentil samples ^1^ injected two times in four hydrolysis batches on two different matrices (lentil and BSA) analysed over 4 weeks.

Sample	Hydrolysis 1 (Week 1)		Hydrolysis 2 (Week 2)		Hydrolysis 3 (Week 3)		Hydrolysis 4 (Week 4)			
	BSA (*n* = 2)	Lentil (*n* = 2)	BSA (*n* = 2)	Lentil (*n* = 2)	BSA (*n* = 2)	Lentil (*n* = 2)	BSA (*n* = 2)	Lentil (*n* = 2)	Overall RSD: BSA	Overall RSD: Lentil
Lysine	6.0%	0.9%	7.9%	2.8%	10.5%	1.6%	3.4%	0.4%	8.7%	12.2%
Histidine	9.5%	21.8%	12.0%	15.6%	9.2%	3.8%	5.6%	1.3%	11.6%	15.6%
Cystine *	27.5%	-	39.3%	-	7.9%	-	-	-	47.7%	-
Arginine	0.7%	4.3%	2.4%	1.0%	2.4%	3.2%	5.1%	0.4%	6.6%	10.3%
Glycine	2.2%	0.7%	8.6%	2.1%	2.1%	3.2%	1.0%	3.9%	18.4%	9.1%
Serine	2.8%	3.6%	6.9%	0.6%	8.7%	6.9%	5.7%	0.1%	16.6%	17.0%
Aspartic acid	2.9%	2.6%	2.1%	1.3%	2.0%	2.4%	1.7%	4.1%	16.3%	23.2%
Alanine	1.9%	1.1%	0.4%	4.3%	10.1%	1.1%	6.3%	5.4%	14.7%	14.0%
Threonine	3.7%	1.8%	1.1%	4.8%	8.4%	1.3%	6.4%	3.9%	12.0%	13.0%
Glutamic acid	9.2%	4.2%	3.1%	4.1%	5.7%	6.3%	9.4%	2.5%	13.0%	7.6%
Proline	8.8%	1.0%	2.2%	2.9%	6.3%	7.2%	23.3%	2.9%	16.5%	11.7%
Valine	5.6%	0.9%	1.3%	2.2%	4.7%	5.6%	32.0%	2.6%	21.7%	17.0%
Methionine	4.8%	0.8%	2.1%	1.4%	15.4%	4.0%	16.6%	3.7%	41.5%	55.4%
Isoleucine	0.9%	1.7%	0.9%	3.2%	5.8%	8.0%	17.1%	5.2%	12.7%	14.3%
Leucine	10.2%	0.4%	2.3%	1.1%	1.2%	1.3%	9.7%	6.5%	17.2%	23.4%
Tyrosine	4.3%	1.5%	2.9%	2.4%	4.2%	4.2%	18.3%	2.8%	12.3%	8.3%
Phenylalanine	7.0%	1.2%	3.1%	2.7%	5.9%	1.9%	13.2%	1.0%	7.9%	7.2%
Tryptophan	6.4%	-	2.3%	-	7.3%	-	18.4%	-	11.3%	-
Total protein (mg/g)	6.6%	1.0%	2.3%	2.0%	4.9%	3.7%	8.2%	3.5%	9.7%	12.2%
Valine-d (IS ug/mL)	4.7%	0.4%	1.0%	0.5%	1.0%	3.4%	15.5%	0.3%	14.5%	5.0%

^1^ Supplementary Tables S4 and S5. RSD (%): reproducibility of amino acid content was calculated from four separate hydrolysis batches of BSA and lentil samples. * Cysteine was detected as cystine.

**Table 4:** The average relative standard deviations (RSD) of the peak area ^1^ in a sample injected two times in four hydrolysis batches on two different matrices (lentil and BSA) analysed over 4 weeks.

Sample	Hydrolysis 1 (Week 1)		Hydrolysis 2 (Week 2)		Hydrolysis 3 (Week 3)		Hydrolysis 4 (Week 4)			
	BSA (*n* = 2)	Lentil (*n* = 2)	BSA (*n* = 2)	Lentil (*n* = 2)	BSA (*n* = 2)	Lentil (*n* = 2)	BSA (*n* = 2)	Lentil (*n* = 2)	Overall RSD: BSA	Overall RSD: Lentil
Lysine	6.0%	0.9%	7.9%	2.8%	10.5%	1.6%	3.4%	0.4%	8.9%	5.5%
Histidine	9.5%	21.8%	12.0%	15.6%	9.2%	3.8%	5.6%	1.3%	9.1%	13.4%
Cystine *	27.5%	-	39.3%	-	7.9%	-	-	0.4%	24.9%	-
Arginine	0.7%	4.3%	2.4%	1.0%	2.4%	3.2%	5.1%	3.9%	8.7%	12.3%
Glycine	2.2%	0.7%	8.6%	2.1%	2.1%	3.2%	1.0%	0.1%	7.3%	4.9%
Serine	2.8%	3.6%	6.9%	0.6%	8.7%	6.9%	5.6%	3.7%	8.9%	10.6%
Aspartic acid	2.7%	2.3%	2.1%	1.3%	2.0%	2.4%	1.6%	5.4%	8.6%	14.9%
Alanine	1.9%	1.1%	0.4%	4.3%	10.1%	1.1%	6.3%	3.9%	7.7%	7.2%
Threonine	3.7%	1.8%	1.1%	4.8%	8.4%	1.3%	6.4%	1.9%	5.1%	6.0%
Glutamic acid	7.7%	3.3%	2.2%	2.3%	4.0%	3.3%	7.7%	1.7%	8.0%	5.5%
Proline	6.6%	0.7%	1.4%	1.6%	3.7%	3.9%	15.2%	1.7%	10.7%	7.5%
Valine	3.8%	0.6%	0.8%	1.3%	2.4%	4.1%	15.7%	0.3%	9.0%	8.8%
Valine-d (IS ug/mL)	4.7%	0.4%	1.0%	0.5%	1.0%	3.4%	15.5%	3.7%	8.0%	9.4%
Methionine	4.8%	0.8%	2.1%	1.4%	15.4%	4.0%	16.6%	5.2%	34.9%	43.8%
Isoleucine	0.9%	1.7%	0.8%	2.4%	5.1%	6.5%	17.1%	7.2%	10.7%	11.6%
Leucine	4.7%	0.3%	2.2%	1.0%	1.2%	1.3%	7.6%	2.8%	7.8%	5.5%
Tyrosine	4.3%	1.5%	2.9%	2.4%	4.2%	4.2%	18.3%	1.0%	12.4%	8.5%
Phenylalanine	7.0%	1.2%	3.1%	2.7%	5.9%	1.9%	13.2%	2.6%	14.2%	7.1%
Tryptophan	6.4%	<LoQ	2.3%	<LoQ	7.3%	<LoQ	18.4%	0.4%	11.8%	-
Average	4.8%	2.8%	3.4%	2.8%	5.8%	3.3%	10.0%	1.3%	10.7%	10.7%

^1^Supplementary Tables S6 and S7. RSD (%): reproducibility of peak area was calculated from four separate hydrolysis batches of BSA and lentil samples. * Cysteine was detected as cystine.

**Table 5:** The average relative standard deviations (RSD) of retention time ^1^ of BSA and lentil samples injected two times in four hydrolysis batches on two different matrices (lentil and BSA) analysed over 4 weeks.

Sample Name No. of Injections	BSA 1 (*n* = 2)	BSA 2 (*n* = 2)	BSA 3 (*n* = 2)	BSA 4 (*n* = 2)	QC 1 (*n* = 2)	QC 2 (*n* = 2)	QC 3 (*n* = 2)	QC 4 (*n* = 2)	Overall RSD (%)
Lysine	0.0%	0.1%	0.1%	0.1%	0.1%	0.1%	0.0%	0.2%	0.2%
Histidine	0.1%	0.0%	0.0%	0.3%	0.1%	0.0%	0.0%	0.1%	0.1%
Cystine *	0.3%	0.1%	0.1%	-	-	-	-	0.2%	0.2%
Arginine	0.0%	0.0%	0.1%	0.1%	0.1%	0.0%	0.0%	0.1%	0.1%
Glycine	0.2%	0.0%	0.1%	0.3%	0.1%	0.0%	0.0%	0.1%	0.1%
Serine	0.2%	0.0%	0.1%	0.3%	0.1%	0.0%	0.0%	0.1%	0.1%
Aspartic acid	0.2%	0.0%	0.1%	0.0%	0.1%	0.0%	0.2%	0.2%	0.2%
Alanine	0.0%	0.0%	0.1%	0.2%	0.1%	0.0%	0.0%	0.1%	0.1%
Threonine	0.2%	0.2%	0.1%	0.0%	0.1%	0.0%	0.0%	0.1%	0.1%
Glutamic acid	0.0%	0.0%	0.1%	0.2%	0.1%	0.0%	0.0%	0.2%	0.2%
Proline	0.1%	0.1%	0.1%	0.2%	0.1%	0.2%	0.0%	0.1%	0.1%
Valine	0.1%	0.1%	0.1%	0.0%	0.1%	0.0%	0.1%	0.1%	0.1%
Valine-d (IS ug/mL)	0.1%	0.1%	0.1%	0.0%	0.1%	0.0%	0.1%	0.1%	0.1%
Methionine	0.0%	0.0%	0.0%	0.1%	0.0%	0.1%	0.0%	0.2%	0.2%
Isoleucine	0.0%	0.1%	0.0%	0.2%	0.2%	0.1%	0.1%	0.1%	0.1%
Leucine	0.0%	0.1%	0.2%	0.3%	0.0%	0.2%	0.1%	0.2%	0.2%
Tyrosine	0.1%	0.1%	0.6%	0.1%	0.0%	0.1%	0.0%	0.7%	0.7%
Phenylalanine	0.1%	0.3%	0.2%	0.3%	0.4%	0.4%	0.0%	0.4%	0.4%
Tryptophan	0.1%	0.0%	0.0%	0.0%	0.0%	0.1%	0.2%	0.2%	0.2%

^1^Supplementary Table S8. RT: retention time; RSD (%): reproducibility of RT was calculated from four separate hydrolysis batches of BSA and lentil samples. * Cysteine was detected as cystine.

## Data Availability

The datasets generated during the current study are available from the corresponding author on reasonable request.

## Supplementary Materials

The following are available online, Figure S1: EIC of 20 amino acids (standards); Table S1: Concentration of amino acids as measured by LC–MS with four hydrolysis methods of a lentil QC 1 (*n* = 5); Table S2: Concentration of amino acids as measured by LC–MS with four hydrolysis methods of lentil QC 2 (*n* = 5); Table S3: Concentration of amino acids as measured by LC–MS with four hydrolysis methods of a BSA sample (*n* = 2); Table S4: Repeatability (RSD in %) and reproducibility (RSD in %) of amino acid (mg/g) content in hydrolysis batch 1–4 for BSA; Table S5: Repeatability (RSD in %) and reproducibility (RSD in %) of amino acid (mg/g) content in hydrolysis batch 1–4 for lentil (QC); Table S6: Peak area repeatability (RSD in %) and reproducibility (RSD in %) in hydrolysis batch 1–4 for BSA samples; Table S7: Peak area repeatability (RSD in %) and reproducibility (RSD in %) in hydrolysis batch 1–4 for lentil samples QC 1–QC 4; Table S8: Retention time (min) repeatability (RSD in %) and reproducibility (RSD in %) methodology in hydrolysis batch 1–4 BSA and QC (lentil) sample.

## Sample Availability

Samples and standards are available from the authors.
