# Supplementary material for: High-Throughput Analysis of Amino Acids for Protein Quantification in Plant and Animal-Derived Samples Using High Resolution Mass Spectrometry
Source: Molecules. 2021 Dec 14;26(24):7578. doi: 10.3390/molecules26247578 (PMC8705696; doi:10.3390/molecules26247578)
Supplement: Supplementary file 1 [file molecules-26-07578-s001.zip › molecules-1455607-supplementary.pdf]

High-throughput analysis of amino acids for protein quantification in plant and animal-derived samples using high resolution mass spectrometry

Priyanka Reddy [1], Aaron Elkins [1], Joe Panozzo [1] and Simone J Rochfort [1,2*]

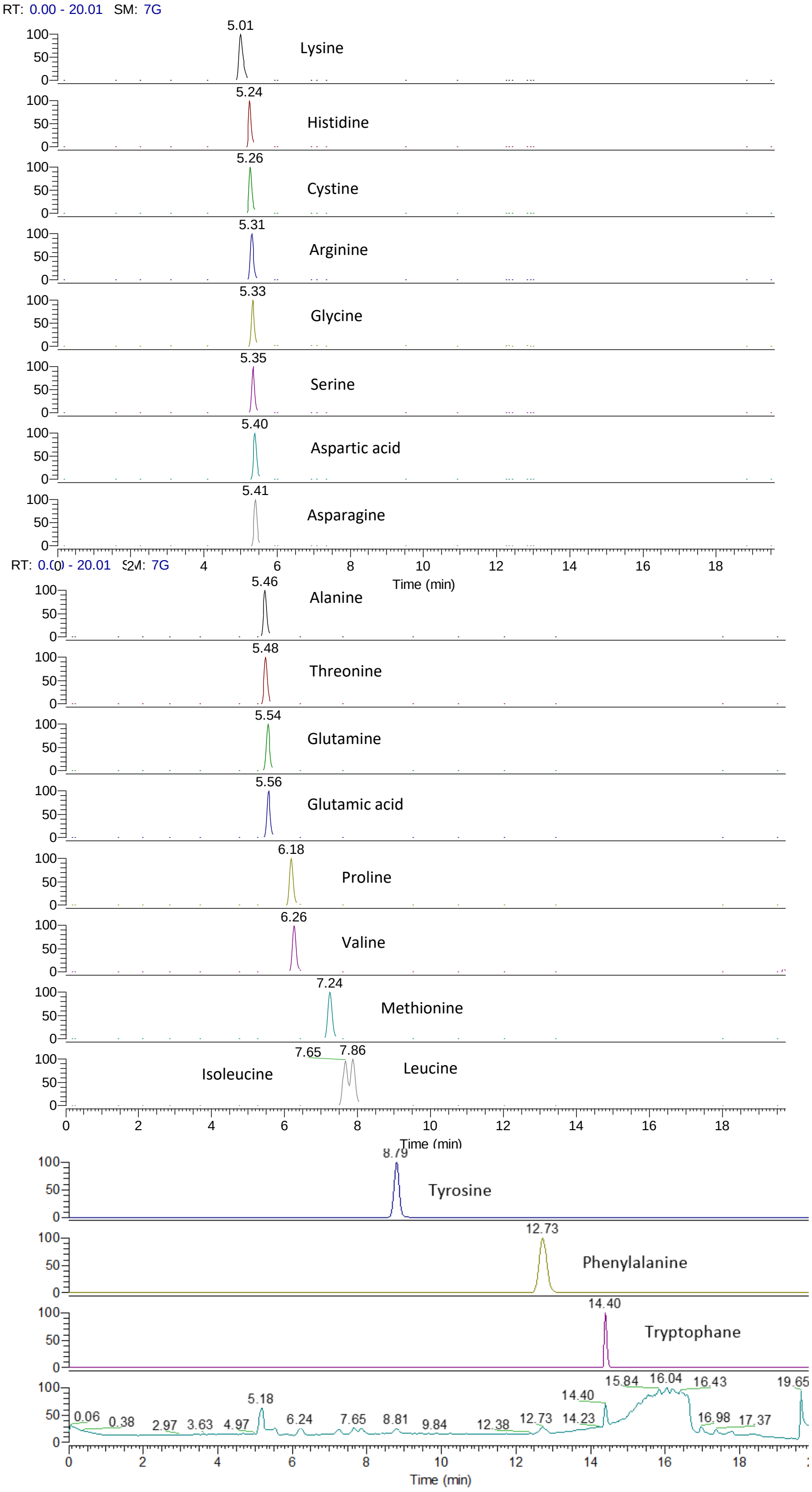

Figure S1. EIC and TIC of 20 amino acids (standards).

Table S1. Concentration of amino acids as measured by LCMS with four hydrolysis methods of a lentil QC 1 (n= 5).

Analyte	4 M MetS + 0.2% tryptamine : 22 hr at 110°C			4 M MetS + 0.2% tryptamine: 60 min 150°C			6 M HCl + 0.2% tryptamine: 22 hr at 110°C			6 M HCl + 0.2% tryptamine : 60 min at 150°C		
**Lysine**	1.23	±	0.01	1.02	±	0.07	1.76	±	0.22	1.52	±	0.09
**Histidine**	0.25	±	0.01	0.22	±	0.01	0.16	±	0.00	0.13	±	0.01
**Arginine**	0.63	±	0.01	0.60	±	0.02	1.19	±	0.03	0.62	±	0.14
**Serine**	0.16	±	0.00	0.11	±	0.01	0.03	±	0.03	0.14	±	0.04
**Aspartic acid**	1.17	±	0.03	0.72	±	0.05	0.10	±	0.09	1.11	±	0.05
**Glycine**	34.12	±	7.74	20.62	±	4.16	17.26	±	5.76	26.31	±	4.06
**Alanine**	1.15	±	0.02	1.04	±	0.02	1.70	±	0.42	1.55	±	0.12
**Threonine**	1.07	±	0.01	0.92	±	0.02	0.84	±	0.20	0.88	±	0.05
**Glutamic acid**	8.43	±	0.05	7.91	±	0.15	4.05	±	1.14	6.56	±	0.08
**Cystine***	2.88×10^{-2}	±	1.8×10^{-2}	0	±	0	5.63×10^{-4}	±	2.16×10^{-4}	0.12	±	0.03
**Proline**	763.47	±	2.47	714.59	±	14.73	571.70	±	25.60	473.73	±	17.05
**Valine**	7.45	±	0.14	6.20	±	0.18	6.99	±	0.22	4.51	±	0.14
**Methionine**	1.05	±	0.09	1.44	±	0.10	1.30	±	0.03	0.82	±	0.04
**Isoleucine**	8.82	±	0.29	7.16	±	0.37	8.11	±	0.11	4.80	±	0.16
**Leucine**	16.47	±	0.28	14.38	±	0.31	15.53	±	0.24	8.87	±	0.09
**Tyrosine**	8.05	±	0.09	4.86	±	0.18	5.45	±	0.06	3.53	±	0.09
**Phenylalanine**	12.83	±	0.20	11.10	±	0.16	11.77	±	0.24	6.50	±	0.19
**Tryptophan**	0.01	±	0.00	0.01	±	0.00	0.01	±	0.00	0.01	±	9.0×10^{-5}
**Total protein**	**866.40**	**±**	**11.46**	**792.91**	**±**	**20.53**	**647.94**	**±**	**34.41**	**541.73**	**±**	**22.41**

*Cysteine was detected as cystine.

Table S2. Concentration of amino acids as measured by LCMS with four hydrolysis methods of lentil QC 2 (n= 5).

Analyte	4 M MetS + 0.2% tryptamine : 22 hr at 110°C			4 M MetS + 0.2% tryptamine: 60 min 150°C			6 M HCl + 0.2% tryptamine: 22 hr at 110°C			6 M HCl + 0.2% tryptamine : 60 min at 150°C		
**Lysine**	1.15	±	0.01	0.97	±	0.03	1.38	±	0.02	1.24	±	0.02
**Histidine**	0.23	±	0.01	0.20	±	0.01	0.24	±	0.01	0.21	±	0.00
**Arginine**	0.59	±	0.01	0.62	±	0.01	1.41	±	0.03	1.35	±	0.02
**Serine**	0.15	±	0.00	0.14	±	0.00	0.21	±	0.02	0.22	±	0.01
**Aspartic acid**	0.93	±	0.01	0.92	±	0.02	0.98	±	0.12	1.04	±	0.05
**Glycine**	8.48	±	1.25	7.83	±	1.32	12.78	±	0.76	11.84	±	0.54
**Alanine**	0.98	±	0.02	0.97	±	0.03	2.61	±	0.03	2.56	±	0.03
**Threonine**	0.94	±	0.00	0.79	±	0.01	1.19	±	0.02	1.13	±	0.01
**Glutamic acid**	7.82	±	0.06	7.47	±	0.12	6.84	±	0.10	6.84	±	0.05
**Cystine***	$3.24x10^{-3}$	±	$7.8x10^{-4}$	$1.7x10^{-3}$	±	$2.7x10^{-4}$	ND			$1.1x10^{-4}$	±	$3.0x10^{-5}$
**Proline**	812.77	±	3.51	779.89	±	14.11	744.09	±	5.69	718.11	±	3.18
**Valine**	6.14	±	0.09	4.35	±	0.19	6.79	±	0.03	5.99	±	0.09
**Methionine**	0.58	±	0.04	1.31	±	0.08	0.67	±	0.10	1.19	±	0.03
**Isoleucine**	7.66	±	0.10	5.40	±	0.22	8.88	±	0.05	7.52	±	0.20
**Leucine**	12.88	±	0.11	11.28	±	0.28	14.20	±	0.13	13.21	±	0.18
**Tyrosine**	7.33	±	0.07	6.36	±	0.17	5.92	±	0.14	6.13	±	0.06
**Phenylalanine**	11.22	±	0.14	9.64	±	0.24	11.72	±	0.07	10.88	±	0.08
**Tryptophan**	<LOQ			<LOQ			<LOQ			<LOQ		
**Total protein**	**868.62**	±	**5.28**	**828.52**	±	**16.62**	**808.18**	±	**7.25**	**778.57**	±	**4.46**

*Cysteine was detected as cystine.

Table S3. Concentration of amino acids as measured by LCMS with four hydrolysis methods of a BSA sample (n= 2).

Analyte	4 M MetS + 0.2% tryptamine at 110°C			4 M MetS + 0.2% tryptamine at 150°C			6 M HCl + 0.2% tryptamine at 110°C			6 M HCl + 0.2% tryptamine at 150°C		
**Lysine**	1.79	±	0.14	1.78	±	0.10	2.13	±	0.35	2.32	±	0.20
**Histidine**	0.33	±	0.05	0.28	±	0.01	0.26	±	0.00	0.24	±	0.09
**Arginine**	0.31	±	0.02	0.29	±	0.01	0.63	±	0.04	0.53	±	0.28
**Serine**	0.08	±	0.01	0.07	±	0.02	0.07	±	0.07	0.08	±	0.05
**Aspartic acid**	0.77	±	0.06	0.68	±	0.09	0.41	±	0.38	0.83	±	0.02
**Glycine**	7.47	±	0.32	7.55	±	0.57	5.10	±	0.53	6.99	±	2.44
**Alanine**	1.16	±	0.08	1.16	±	0.02	2.71	±	0.25	2.40	±	0.95
**Threonine**	1.24	±	0.05	1.16	±	0.03	1.32	±	0.10	1.30	±	0.32
**Glutamic acid**	6.92	±	0.18	6.74	±	0.24	4.82	±	0.67	5.52	±	0.69
**Cystine***	$3.59x10^{-1}$	±	$3.58x10^{-1}$	ND	±	-	$1.01x10^{-3}$	±	$7.84x10^{-5}$	$4.74x10^{-3}$	±	$4.46x10^{-3}$
**Proline**	753.31	±	1.74	750.01	±	28.31	635.81	±	61.23	610.19	±	165.59
**Valine**	7.04	±	0.93	6.07	±	0.83	6.82	±	0.31	5.80	±	1.11
**Methionine**	0.68	±	0.08	1.50	±	0.22	1.23	±	0.09	0.93	±	0.31
**Isoleucine**	4.71	±	0.44	4.03	±	0.76	4.66	±	0.10	3.52	±	0.90
**Leucine**	17.60	±	1.31	16.41	±	2.12	16.80	±	0.69	13.80	±	3.21
**Tyrosine**	10.57	±	0.30	9.88	±	0.53	8.95	±	0.02	7.80	±	2.65
**Phenylalanine**	13.40	±	0.44	12.11	±	0.56	12.62	±	0.37	10.02	±	3.32
**Tryptophan**	1.36	±	0.02	1.35	±	0.001	ND		-	0.01	±	0.00183
**Total protein**	**829.12**	**±**	**2.23**	**818.67**	**±**	**24.86**	**701.97**	**±**	**63.43**	**672.29**	**±**	**176.84**

*Cysteine was detected as cystine.

Supplementary Table S4. Repeatability (RSD in %) and reproducibility (RSD in %) of amino acid (mg/g) content in hydrolysis batch 1-4 for BSA.

	**Hydrolysis 1**			**Hydrolysis 2**			**Hydrolysis 3**			**Hydrolysis 4**			
**Sample**	BSA 1a	BSA 1b	**RSD (%)**	BSA 2a	BSA 2b	**RSD (%)**	BSA 3a	BSA 3b	**RSD (%)**	BSA 4a	BSA 4b	**RSD (%)**	**Overall Reproducibility RSD (%)**
**wt (mg)**	10.3	10.3		10.2	10.2		10.2	10.2		10.2	10.2		
**Lysine**	21.8	23.7	6.0%	23.0	20.6	7.9%	23.6	20.4	10.5%	24.8	26.0	3.4%	**8.7%**
**Histidine**	10.6	9.3	9.5%	7.2	8.6	12.0%	9.1	8.0	9.2%	8.2	8.9	5.6%	**11.6%**
**Cystine***	0.6	0.90	27.5%	0.4	0.3	39.3%	0.3	0.3	7.9%	0.4	-	-	**47.7%**
**Arginine**	5.9	6.0	0.7%	5.1	5.3	2.4%	5.6	5.4	2.4%	5.8	6.2	5.1%	**6.6%**
**Glycine**	0.9	0.9	2.2%	0.7	0.6	8.6%	0.6	0.6	2.1%	0.8	0.8	1.0%	**18.4%**
**Serine**	1.9	1.9	2.8%	1.5	1.4	6.9%	1.3	1.2	8.7%	1.7	1.6	5.7%	**16.6%**
**Aspartic acid**	7.4	7.7	2.9%	5.7	5.9	2.1%	5.6	5.4	2.0%	7.7	7.8	1.7%	**16.3%**
**Alanine**	7.2	7.4	1.9%	6.2	6.1	0.4%	6.1	5.3	10.1%	7.6	8.3	6.3%	**14.7%**
**Threonine**	8.8	9.3	3.7%	7.4	7.2	1.1%	7.7	6.9	8.4%	8.6	9.4	6.4%	**12.0%**
**Glutamic acid**	38.1	43.4	9.2%	37.6	35.9	3.1%	39.0	35.9	5.7%	44.7	51.0	9.4%	**13.0%**
**Proline**	34.1	38.6	8.8%	35.9	34.8	2.2%	38.5	35.3	6.3%	38.7	53.9	23.3%	**16.5%**
**Valine**	42.9	46.4	5.6%	39.7	38.9	1.3%	47.2	44.2	4.7%	44.8	71.1	32.0%	**21.7%**
**Methionine**	4.8	5.2	4.8%	4.1	4.0	2.1%	1.8	1.4	15.4%	5.1	6.4	16.6%	**41.5%**
**Isoleucine**	21.7	21.4	0.9%	18.1	17.9	0.9%	21.6	19.9	5.8%	20.7	26.4	17.1%	**12.7%**
**Leucine**	109.5	126.5	10.2%	82.3	79.7	2.3%	87.1	85.6	1.2%	98.7	86.1	9.7%	**17.2%**

**Tyrosine**	47.4	50.4	4.3%	45.7	43.9	2.9%	49.3	46.5	4.2%	49.1	63.6	18.3%	**12.3%**
**Phenylalanine**	54.4	60.1	7.0%	54.6	52.2	3.1%	60.1	55.3	5.9%	56.2	46.7	13.2%	**7.9%**
**Tryptophan**	4.7	5.1	6.4%	5.0	4.8	2.3%	5.3	4.7	7.3%	5.0	6.5	18.4%	**11.3%**
**Total protein (mg/g)**	**422.2**	**463.2**	**6.6%**	**379.7**	**367.8**	**2.3%**	**409.4**	**381.8**	**4.9%**	**428.0**	**480.7**	**8.2%**	**9.7%**
**Valine-d (IS ug/mL)**	0.81	0.87	4.7%	0.69	0.70	1.0%	0.71	0.70	1.0%	0.82	1.02	15.5%	**14.5%**

*Cysteine was detected as cystine.

Supplementary Table S5. Repeatability (RSD in %) and reproducibility (RSD in %) of amino acid (mg/g) content in hydrolysis batch 1-4 for lentil (QC).

	**Hydrolysis 1**			**Hydrolysis 2**				**Hydrolysis 3**				**Hydrolysis 4**			
**Sample**	QC 1a	QC 1b	**RSD (%)**	QC 2a	QC 2b	QC 2c	**RSD (%)**	QC 3a	QC 3b	QC 3c	**RSD (%)**	QC 4a	QC 4b	**RSD (%)**	**Overall Reproducibility RSD (%)**
**wt (mg)**	50.1	50.1		50.0	50.0	50.0		50.2	50.2	50.2		50.2	50.2		
**Lysine**	3.3	3.2	0.9%	2.7	2.6	2.7	2.8%	2.7	2.8	2.7	1.6%	3.5	3.5	0.4%	**12.2%**
**Histidine**	1.9	1.4	21.8%	1.4	1.1	1.1	15.6%	1.3	1.3	1.2	3.8%	1.4	1.4	1.3%	**15.6%**
**Arginine**	2.2	2.4	4.3%	1.9	1.9	1.9	1.0%	2.0	2.0	1.9	3.2%	2.3	2.3	0.4%	**10.3%**
**Glycine**	0.5	0.5	0.7%	0.4	0.4	0.4	2.1%	0.4	0.4	0.4	3.2%	0.4	0.5	3.9%	**9.1%**
**Serine**	0.6	0.6	3.6%	0.4	0.4	0.4	0.6%	0.5	0.5	0.4	6.9%	0.6	0.6	0.1%	**17.0%**
**Aspartic acid**	2.0	2.1	2.6%	1.3	1.3	1.3	1.3%	1.6	1.6	1.6	2.4%	2.3	2.1	4.1%	**23.2%**
**Alanine**	1.3	1.4	1.1%	1.1	1.1	1.0	4.3%	1.1	1.1	1.1	1.1%	1.6	1.4	5.4%	**14.0%**
**Threonine**	1.5	1.5	1.8%	1.2	1.2	1.1	4.8%	1.3	1.3	1.2	1.3%	1.6	1.5	3.9%	**13.0%**
**Glutamic acid**	10.8	10.2	4.2%	10.6	10.9	10.0	4.1%	11.1	11.6	10.3	6.3%	12.5	12.1	2.5%	**7.6%**
**Proline**	8.2	8.1	1.0%	8.0	7.6	7.7	2.9%	8.7	8.5	7.6	7.2%	10.4	10.0	2.9%	**11.7%**
**Valine**	8.4	8.3	0.9%	8.2	7.9	7.9	2.2%	6.1	6.2	5.6	5.6%	9.5	9.2	2.6%	**17.0%**

**Methionine**	1.0	1.0	0.8%	0.3	0.3	0.3	1.4%	0.5	0.5	0.5	4.0%	1.2	1.1	3.7%	**55.4%**
**Isoleucine**	7.8	7.6	1.7%	8.5	7.9	8.2	3.2%	6.4	5.9	5.5	8.0%	8.4	7.8	5.2%	**14.3%**
**Leucine**	16.7	16.8	0.4%	14.1	13.9	13.8	1.1%	12.8	13.1	13.0	1.3%	23.4	21.4	6.5%	**23.4%**
**Tyrosine**	7.4	7.2	1.5%	7.0	6.6	6.8	2.4%	6.8	6.7	6.3	4.2%	8.2	7.8	2.8%	**8.3%**
**Phenylalanine**	10.3	10.5	1.2%	10.0	10.0	9.6	2.7%	9.0	9.1	8.8	1.9%	10.7	10.5	1.0%	**7.2%**
**Tryptophan**	<LOQ	<LOQ	-	<LOQ	<LOQ	<LOQ	-	<LOQ	<LOQ	<LOQ	-	<LOQ	<LOQ	-	**-**
**Total protein (mg/g)**	**83.9**	**82.7**	**1.0%**	**77.1**	**75.1**	**74.3**	**2.0%**	**72.2**	**72.6**	**68.0**	**3.7%**	**98.0**	**93.4**	**3.5%**	**12.2%**
**Valine-d (IS ug/mL)**	0.82	0.81	**0.4%**	0.79	0.78	0.79	**0.5%**	0.77	0.79	0.74	**3.4%**	0.87	0.86	**0.3%**	5.0%

Supplementary Table S6. Peak area repeatability (RSD in %) and reproducibility (RSD in %) in hydrolysis batch 1-4 for BSA samples.

Sample Name	Hydrolysis 1			Hydrolysis 2			Hydrolysis 3			Hydrolysis 4			Overall Reproducibility RSD (%)
	BSA 1a	BSA 1b		BSA 2a	BSA 2b		BSA 3a	BSA 3b		BSA 4a	BSA 4b		
	Peak area		RSD (%)	Peak area		RSD (%)	Peak area		RSD (%)	Peak area		RSD (%)	
Lysine	8.18E+07	8.90E+07	6.0%	1.05E+08	9.35E+07	7.9%	1.07E+08	9.25E+07	10.5%	9.71E+07	1.02E+08	3.4%	8.9%
Histidine	4.48E+07	3.92E+07	9.5%	3.33E+07	3.94E+07	12.0%	4.17E+07	3.66E+07	9.2%	3.95E+07	4.27E+07	5.6%	9.1%
Cystine*	2.05E+06	4.48E+07	27.5%	1.63E+06	9.19E+05	39.3%	1.28E+06	1.15E+06	7.9%	1.40E+06	-	-	24.9%
Arginine	1.09E+08	1.10E+08	0.7%	9.22E+07	9.54E+07	2.4%	1.00E+08	9.70E+07	2.4%	1.10E+08	1.18E+08	5.1%	8.7%
Glycine	1.12E+06	1.08E+06	2.2%	1.09E+06	9.65E+05	8.6%	9.47E+05	9.19E+05	2.1%	1.05E+06	1.07E+06	0.9%	7.3%
Serine	8.46E+06	8.13E+06	2.8%	8.25E+06	7.48E+06	6.9%	7.23E+06	6.40E+06	8.7%	8.16E+06	7.53E+06	5.6%	8.9%
Aspartic acid	3.82E+07	3.96E+07	2.7%	3.44E+07	3.55E+07	2.1%	3.36E+07	3.26E+07	2.0%	3.99E+07	4.08E+07	1.6%	8.6%
Alanine	3.74E+07	3.85E+07	1.9%	4.19E+07	4.17E+07	0.4%	4.18E+07	3.63E+07	10.1%	4.20E+07	4.59E+07	6.3%	7.7%
Threonine	6.77E+07	7.13E+07	3.7%	6.97E+07	6.86E+07	1.1%	7.31E+07	6.49E+07	8.4%	7.02E+07	7.68E+07	6.4%	5.1%
Glutamic acid	3.19E+08	3.56E+08	7.7%	3.37E+08	3.26E+08	2.2%	3.45E+08	3.26E+08	4.0%	3.63E+08	4.04E+08	7.7%	8.0%
Proline	7.92E+08	8.70E+08	6.6%	1.01E+09	9.88E+08	1.4%	1.05E+09	9.96E+08	3.7%	8.85E+08	1.10E+09	15.2%	10.7%
Valine	7.58E+08	8.00E+08	3.8%	8.44E+08	8.35E+08	0.8%	9.29E+08	8.98E+08	2.4%	7.88E+08	9.85E+08	15.7%	9.0%

**Valine-d (IS ug/mL)**	6.84E+07	7.31E+07	**4.7%**	7.65E+07	7.76E+07	**1.0%**	7.77E+07	7.67E+07	**1.0%**	7.14E+07	8.90E+07	**15.5%**	**8.0%**
**Methionine**	8.49E+07	9.08E+07	**4.8%**	9.39E+07	9.11E+07	**2.1%**	4.10E+07	3.30E+07	**15.4%**	9.01E+07	1.14E+08	**16.6%**	**34.9%**
**Isoleucine**	5.82E+08	5.75E+08	**0.9%**	6.35E+08	6.28E+08	**0.8%**	7.41E+08	6.89E+08	**5.1%**	5.81E+08	7.41E+08	**17.1%**	**10.7%**
**Leucine**	2.52E+09	2.69E+09	**4.7%**	2.86E+09	2.77E+09	**2.2%**	3.02E+09	2.97E+09	**1.2%**	2.67E+09	2.40E+09	**7.6%**	**7.8%**
**Tyrosine**	7.34E+08	7.80E+08	**4.3%**	9.39E+08	9.01E+08	**2.9%**	1.01E+09	9.54E+08	**4.2%**	7.79E+08	1.01E+09	**18.3%**	**12.4%**
**Phenylalanine**	1.58E+09	1.74E+09	**7.0%**	1.99E+09	1.90E+09	**3.1%**	2.19E+09	2.02E+09	**5.9%**	1.70E+09	1.41E+09	**13.2%**	**14.2%**
**Tryptophan**	1.40E+08	1.53E+08	**6.4%**	1.82E+08	1.76E+08	**2.3%**	1.91E+08	1.72E+08	**7.3%**	1.52E+08	1.97E+08	**18.4%**	**11.8%**
**Average**			**4.8%**			**3.4%**			**5.8%**			**10.0%**	**10.7%**

Supplementary Table S7. Peak area repeatability (RSD in %) and reproducibility (RSD in %) in hydrolysis batch 1-4 for lentil samples QC1 -QC4.

	**Hydrolysis 1**			**Hydrolysis 2**				**Hydrolysis 3**				**Hydrolysis 4**			
**Sample Name**	QC 1a	QC 1b		QC 2a	QC 2b	QC 2c		QC 3a	QC 3b	QC 3c		QC 4a	QC 4b		**Overall Reproducibility RSD (%)**
	**Peak area**		**RSD (%)**	**Peak area**			**RSD (%)**	**Peak area**			**RSD (%)**	**Peak area**		**RSD (%)**	
**Lysine**	5.96E+07	5.89E+07	**0.9%**	6.04E+07	5.78E+07	6.09E+07	**2.8%**	6.01E+07	6.20E+07	6.08E+07	**1.6%**	6.78E+07	6.74E+07	**0.4%**	**5.5%**
**Histidine**	3.85E+07	2.82E+07	**21.8%**	3.24E+07	2.57E+07	2.53E+07	**15.6%**	3.00E+07	2.92E+07	2.78E+07	**3.8%**	3.31E+07	3.37E+07	**1.3%**	**13.4%**
**Arginine**	2.02E+08	2.14E+08	**4.3%**	1.68E+08	1.65E+08	1.65E+08	**1.0%**	1.76E+08	1.76E+08	1.67E+08	**3.2%**	2.18E+08	2.16E+08	**3.9%**	**12.3%**
**Glycine**	2.84E+06	2.81E+06	**0.7%**	3.16E+06	3.06E+06	3.04E+06	**2.1%**	3.17E+06	3.29E+06	3.09E+06	**3.2%**	2.97E+06	3.14E+06	**0.1%**	**4.9%**
**Serine**	1.22E+07	1.29E+07	**3.6%**	1.05E+07	1.04E+07	1.04E+07	**0.6%**	1.34E+07	1.27E+07	1.17E+07	**6.7%**	1.35E+07	1.35E+07	**3.7%**	**10.6%**
**Aspartic acid**	4.96E+07	5.13E+07	**2.3%**	3.70E+07	3.79E+07	3.72E+07	**1.3%**	4.65E+07	4.82E+07	4.61E+07	**2.4%**	5.64E+07	5.35E+07	**5.4%**	**14.9%**
**Alanine**	3.35E+07	3.40E+07	**1.1%**	3.77E+07	3.74E+07	3.49E+07	**4.3%**	3.83E+07	3.76E+07	3.75E+07	**1.1%**	4.25E+07	3.94E+07	**3.9%**	**7.2%**
**Threonine**	5.60E+07	5.75E+07	**1.8%**	5.62E+07	5.69E+07	5.21E+07	**4.8%**	5.87E+07	5.87E+07	5.74E+07	**1.3%**	6.52E+07	6.17E+07	**1.9%**	**6.0%**

**Glutamic acid**	4.16E+08	3.97E+08	**3.3%**	4.14E+08	4.19E+08	4.01E+08	**2.3%**	4.25E+08	4.34E+08	4.07E+08	**3.2%**	4.68E+08	4.56E+08	**1.7%**	**5.5%**
**Proline**	8.92E+08	8.83E+08	**0.7%**	1.06E+09	1.03E+09	1.04E+09	**1.6%**	1.11E+09	1.10E+09	1.03E+09	**3.8%**	1.07E+09	1.04E+09	**1.7%**	**7.5%**
**Valine**	7.30E+08	7.24E+08	**0.6%**	8.51E+08	8.31E+08	8.33E+08	**1.3%**	7.00E+08	7.12E+08	6.58E+08	**4.1%**	8.10E+08	7.92E+08	**0.3%**	**8.8%**
**Valine-d (IS ug/mL)**	6.89E+07	6.85E+07	**0.4%**	8.72E+07	8.64E+07	8.71E+07	**0.5%**	8.51E+07	8.69E+07	8.12E+07	**3.4%**	7.59E+07	7.56E+07	**3.7%**	**9.4%**
**Methionine**	8.79E+07	8.69E+07	**0.8%**	3.17E+07	3.10E+07	3.09E+07	**1.4%**	5.77E+07	5.65E+07	5.34E+07	**4.0%**	1.01E+08	9.59E+07	**5.2%**	**43.8%**
**Isoleucine**	1.00E+09	9.81E+08	**1.7%**	1.26E+09	1.20E+09	1.22E+09	**2.4%**	1.02E+09	9.59E+08	8.98E+08	**6.3%**	1.16E+09	1.08E+09	**7.2%**	**11.6%**
**Leucine**	2.10E+09	2.11E+09	**0.3%**	2.41E+09	2.37E+09	2.36E+09	**1.0%**	2.20E+09	2.25E+09	2.23E+09	**1.3%**	2.46E+09	2.22E+09	**2.8%**	**5.5%**
**Tyrosine**	5.55E+08	5.44E+08	**1.5%**	7.00E+08	6.68E+08	6.83E+08	**2.4%**	6.85E+08	6.74E+08	6.32E+08	**4.2%**	6.37E+08	6.13E+08	**1.0%**	**8.5%**
**Phenylalanine**	1.46E+09	1.48E+09	**1.2%**	1.79E+09	1.79E+09	1.71E+09	**2.7%**	1.61E+09	1.63E+09	1.57E+09	**1.9%**	1.59E+09	1.56E+09	**2.6%**	**7.1%**
**Tryptophan**	<LOQ	<LOQ		<LOQ	<LOQ	<LOQ		<LOQ	<LOQ	<LOQ		<LOQ	<LOQ	**0.4%**	
**Average**			**2.8%**				**2.8%**				**3.3%**			**1.3%**	**10.7%**

*Cysteine was detected as cystine.

Supplementary Table S8. Retention time (min) repeatability (RSD in %) and reproducibility (RSD in %) methodology in hydrolysis batch 1-4 BSA and QC (lentil) sample.

	**Hydrolysis 1**		**Hydrolysis 2**		**Hydrolysis 3**		**Hydrolysis 4**		**Hydrolysis 1**		**Hydrolysis 2**			**Hydrolysis 3**			**Hydrolysis 4**		
**Sample name**	BSA 1a	BSA 1b	BSA 2a	BSA 2b	BSA 3a	BSA 3b	BSA 4a	BSA 4b	QC 1a	QC 1b	QC 2a	QC 2b	QC 2c	QC 3a	QC 3b	QC 3c	QC 4a	QC 4b	**Overall Reproducibility RSD (%)**
									**RT (min)**										
**Lysine**	5.01	5.01	4.99	4.99	4.98	4.99	5.00	5.00	5.01	5.00	4.99	4.99	4.99	5.00	5.00	4.99	5.00	5.00	**0.17%**
**Histidine**	5.37	5.38	5.38	5.38	5.37	5.38	5.37	5.39	5.37	5.38	5.37	5.38	5.38	5.38	5.37	5.37	5.38	5.37	**0.11%**
**Cystine**	5.38	5.40	5.39	5.38	5.39	5.40	5.40	-	-	-	-	-	-	-	-	-	-	-	**0.17%**
**Arginine**	5.38	5.38	5.38	5.38	5.39	5.40	5.39	5.39	5.39	5.40	5.38	5.38	5.38	5.39	5.38	5.39	5.40	5.39	**0.12%**
**Glycine**	5.38	5.40	5.39	5.39	5.39	5.40	5.39	5.41	5.39	5.40	5.38	5.39	5.39	5.39	5.38	5.39	5.40	5.39	**0.11%**
**Serine**	5.38	5.40	5.39	5.39	5.39	5.40	5.39	5.41	5.40	5.40	5.40	5.39	5.39	5.39	5.38	5.39	5.40	5.39	**0.12%**
**Aspartic acid**	5.46	5.47	5.46	5.45	5.45	5.46	5.47	5.47	5.47	5.46	5.45	5.44	5.44	5.47	5.45	5.46	5.46	5.47	**0.19%**

**Alanine**	5.50	5.50	5.50	5.50	5.50	5.50	5.50	5.51	5.51	5.50	5.49	5.50	5.50	5.50	5.49	5.49	5.50	5.50	**0.11%**
**Threonine**	5.50	5.52	5.52	5.50	5.51	5.52	5.51	5.51	5.51	5.50	5.49	5.50	5.50	5.51	5.51	5.51	5.52	5.51	**0.14%**
**Glutamic acid**	5.58	5.58	5.58	5.58	5.57	5.58	5.57	5.59	5.59	5.58	5.57	5.56	5.56	5.57	5.57	5.57	5.58	5.57	**0.15%**
**Proline**	6.18	6.19	6.18	6.17	6.16	6.17	6.18	6.20	6.18	6.19	6.17	6.18	6.16	6.18	6.18	6.17	6.18	6.18	**0.13%**
**Valine**	6.27	6.27	6.26	6.27	6.26	6.27	6.27	6.27	6.28	6.27	6.27	6.26	6.26	6.27	6.28	6.27	6.28	6.26	**0.11%**
**Valine-d (IS ug/mL)**	6.27	6.27	6.26	6.27	6.26	6.27	6.27	6.27	6.28	6.27	6.27	6.26	6.26	6.27	6.28	6.27	6.28	6.26	**0.11%**
**Methionine**	7.24	7.24	7.23	7.22	7.22	7.22	7.24	7.25	7.25	7.25	7.22	7.23	7.22	7.23	7.23	7.23	7.25	7.23	**0.17%**
**Isoleucine**	7.66	7.66	7.66	7.65	7.66	7.66	7.66	7.68	7.68	7.66	7.64	7.66	7.65	7.68	7.66	7.67	7.67	7.65	**0.13%**
**Leucine**	7.87	7.87	7.87	7.86	7.85	7.87	7.85	7.89	7.87	7.87	7.85	7.87	7.85	7.89	7.87	7.86	7.87	7.86	**0.15%**
**Tyrosine**	8.79	8.80	8.67	8.68	8.67	8.74	8.79	8.80	8.79	8.79	8.66	8.69	8.68	8.68	8.68	8.68	8.81	8.78	**0.67%**
**Phenylalanine**	12.70	12.72	12.64	12.60	12.64	12.61	12.72	12.77	12.79	12.71	12.67	12.67	12.60	12.72	12.64	12.65	12.70	12.69	**0.43%**
**Tryptophan**	14.41	14.40	14.33	14.33	14.33	14.33	14.38	14.39	14.39	14.39	14.33	14.34	14.31	14.32	14.32	14.35	14.39	14.36	**0.24%**
**Average**																			**0.19%**

RT.: retention time; RSD (%): repeatability of RT. was calculated from four separate hydrolysis batches of BSA and lentil samples.
*Cysteine was detected as cystine.
